# First Detection of *Sclerotinia nivalis* on Carrot (*Daucus carota* subsp. *sativus*) in Russia and Comparative Pathogenicity of *Sclerotinia* Isolates on Carrot

**DOI:** 10.3390/plants14223487

**Published:** 2025-11-15

**Authors:** Viktoriya V. Medvedeva, Rashit I. Tarakanov, Peter V. Evseev, Evgenii S. Mazurin, Svetlana I. Chebanenko, Olga O. Beloshapkina, Fevzi S.-U. Dzhalilov, Sokrat G. Monakhos

**Affiliations:** 1 Department of Plant Protection, Russian State Agrarian University—Moscow Timiryazev Agricultural Academy, Timiryazevskaya Str. 49, 127434 Moscow, Russia; vikamedm@mail.ru (V.V.M.); svchebanenko@rgau-msha.ru (S.I.C.); beloshapkina@rgau-msha.ru (O.O.B.); dzhalilov@rgau-msha.ru (F.S.-U.D.); 2 Laboratory of Molecular Microbiology, Pirogov Russian National Research Medical University, Ostrovityanova 1, 117997 Moscow, Russia; petevseev@gmail.com; 3 Laboratory of Technical Support, Syngenta, Letnikovskaya Str. 2, 115114 Moscow, Russia; evgenii.mazurin@syngenta.com; 4 Department of Molecular Breeding, Cell and Seed Technology, Russian State Agrarian University—Moscow Timiryazev Agricultural Academy, Timiryazevskaya Str. 49, 127434 Moscow, Russia; s.monakhos@rgau-msha.ru

**Keywords:** *Sclerotinia nivalis*, *Sclerotinia sclerotiorum*, white mold, molecular identification, pathogenicity, sclerotia, carrot, fungicide sensitivity, fluazinam, first report

## Abstract

White mold of carrot is mainly caused by *Sclerotinia sclerotiorum*, while *Sclerotinia nivalis* is rarely reported. This study provides the first molecular confirmation of *S. nivalis* on carrot in Russia, expanding knowledge of its global distribution. *rDNA-ITS* sequencing (100% identity with reference strains) and phylogenetic analyses confirmed the isolate as *S. nivalis*. The growth, sclerotia formation, temperature response, pathogenicity, and fungicide sensitivity of four *Sclerotinia* strains (*S. sclerotiorum* from carrot, rapeseed, and soybean, and *S. nivalis* from carrot) were compared. *S. nivalis* showed slower growth, smaller but more numerous sclerotia (2–5 mm), and an optimal temperature of 15 °C, lower than *S. sclerotiorum* (25 °C). The soybean strain *S. sclerotiorum* SC382 was the most aggressive, causing 62% necrosis of carrot leaves and complete root decay within 9 days, while *S. nivalis* and the carrot isolates showed moderate aggressiveness. The *S. nivalis* SM8 strain was four times less sensitive to fluazinam (EC_50_ = 0.0107 µg/mL) than *S. sclerotiorum*, whereas sensitivity to boscalid and pyraclostrobin varied. These findings confirm *S. nivalis* as a new causal agent of carrot white mold in Russia and demonstrate the potential of *Sclerotinia* strains from soybean and rapeseed to infect carrot, emphasizing the need for species-level monitoring and adapted control strategies.

## 1. Introduction

Table carrot (*Daucus carota* subsp. *sativus*) is an important root vegetable widely used in human nutrition. Every year, tens of millions of tons are produced worldwide; for example, in Russia, about 1.37 million tons of carrot were harvested in 2023 [1]. However, a significant part of the yield may be lost during storage due to diseases. One of the most destructive diseases of stored carrot is white mold, or sclerotiniosis, caused mainly by the fungus *Sclerotinia sclerotiorum* (Lib.) de Bary [2]. Yield losses from white mold and secondary bacterial infections can reach 50–70% under unfavorable storage conditions [3].

The main causal agent of white mold, *S. sclerotiorum*, is a polyphagous pathogen that persists in soil as sclerotia for up to 5–8 years. Under favorable conditions (humidity above 80%, temperature 10–15 °C), sclerotia germinate carpogenically, forming apothecia that release ascospores dispersed by wind, infecting the aerial organs of carrot [4]. During storage, infection occurs myceliogenically, when sclerotia or mycelium on infected leaf or root residues initiate a new outbreak, especially under conditions of mechanical damage and excessive humidity. On infected tissues, the fungus forms mycelium and new sclerotia, ensuring the survival of the pathogen and its transition to the next growing season [5]. The pathogen has an extremely broad host range and can infect more than 400 plant species under various soil and climatic conditions, including many crops such as soybean, rapeseed, sunflower, and vegetable crops [6]. The expansion of cultivation areas of susceptible crops (rapeseed and soybean) is associated with increased incidence of white mold on carrot [7]. The pathogen forms specialized resting structures called sclerotia, which can persist on plant residues and in soil for several years, serving as infection sources in the field and in storage facilities, creating serious risks when these crops are rotated [4]. In recent years, Russia has seen an increase in the area under soybean and rapeseed cultivation, accompanied by more frequent epiphytotic outbreaks and long-term persistence of sclerotia in agrocenoses. At the same time, the possibility of cross-infection of carrot by *Sclerotinia* isolates from other crops remains insufficiently studied. Although *S. sclerotiorum* is known to cause carrot infection in the field and during storage [3], information on the aggressiveness of isolates from soybean and rapeseed on carrot remains limited [8]. Therefore, assessment of the ability of *Sclerotinia* isolates from rapeseed and soybean to initiate infection on carrot is an important task in phytopathological monitoring. Cross-host infection potential of *Sclerotinia* species is a key challenge for both field production and long-term cold storage, where contaminated plant material from other hosts may serve as a source of primary inoculum.

In addition to *S. sclerotiorum*, carrot rot can also be caused by *Sclerotinia nivalis* [9] and *S. minor* [10], but these species occur less frequently, and their economic impact is usually lower than that of *S. sclerotiorum*. In Russia, the pathogen of carrot white mold has been known as *S. sclerotiorum* [11], and reports of other causal agents of the disease are absent from modern literature.

*Sclerotinia nivalis*, formerly known as *S. intermedia*, was described relatively recently based on morphological features of the sclerotial anamorph and teleomorph obtained in culture as a pathogen of dicotyledonous herbaceous plants [12]. The species was first reported as the causal agent of white mold on ornamental plants, weeds, and carrot (*Daucus carota*) in Japan in 1997 [9]. Further studies demonstrated its wide distribution in the Northern Hemisphere. It can be distinguished by its smaller sclerotia compared to *S. sclerotiorum*, binucleate ascospores, molecular mass of major sclerotial proteins, and esterase isoenzymes in sclerotial extracts [13]. *S. nivalis* is a mesophilic species, with an optimal mycelial growth temperature around 20 °C [12].

The host range of *S. nivalis* includes 84 plant species belonging to 50 genera and 19 families, including carrot (*Daucus carota*) [9], lettuce (*Lactuca sativa*) [14], stringy stonecrop (*Sedum sarmentosum*) [13], hardy kiwi (*Actinidia arguta*) [15], several weeds such as burdock (*Arctium lappa*), common ragweed (*Ambrosia elatior*), ribwort plantain (*Plantago lanceolata*) [9], chrysanthemum (*Chrysanthemum morifolium*), bugleweed (*Ajuga reptans*) [9], japanese angelica (*Angelica acutiloba*) [9], japanese angelica tree (*Aralia elata*) [16], korean pasque flower (*Pulsatilla koreana*) [17,18], Japanese atractylodes (*Atractylodes japonica*) [19], american ginseng (*Panax quinquefolius*) [20], and korean ginseng (*Panax ginseng*) [21]. In Russia, *S. nivalis* was first detected on ornamental and medicinal plants (*Tulipa* sp., *Iris germanica*, *Matricaria inodora*, *Thlaspi arvense*, *Phlox* sp., *Helichrysum arenarium*, *Digitalis purpurea*, *Sedum* sp.) in botanical gardens of Yekaterinburg, Cheboksary, Kirovsk, Vladivostok, Yuzhno-Sakhalinsk, St. Petersburg, and Moscow in 2002 [22,23]. However, infection of carrot by this pathogen in Russia has not been previously reported. Moreover, the identification of *S. nivalis* in those cases was based mainly on morphological criteria (size of sclerotia, binucleate ascospores) and molecular mass of major sclerotial proteins, which is insufficient for precise species identification. Therefore, molecular verification of the white mold pathogen on new hosts is of particular importance.

The purpose of this study was to provide the first molecular identification of *S. nivalis* in carrot samples affected by white mold and to compare the aggressiveness of *Sclerotinia* strains isolated from carrot, rapeseed, and soybean with carrot.

## 2. Results

### 2.1. Isolation and Characterization of Strains

**Figure 1:**
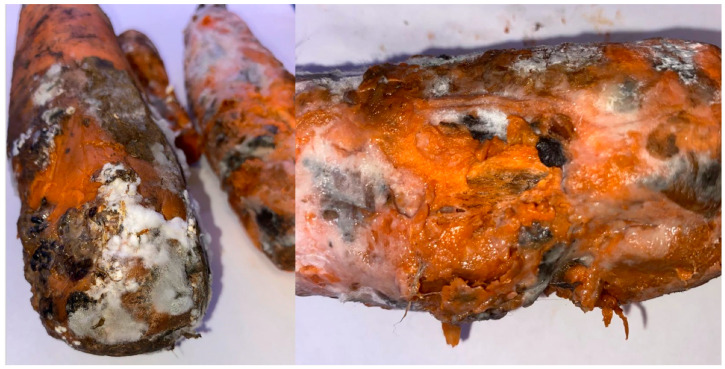
Symptoms of white mold damage to carrot roots in storage at the end of the storage, March 2023, Nizhny Novgorod region, Russia.

In 2023–2024, by the end of the carrot storage period (February–March), during phytosanitary monitoring in vegetable storage facilities of the Nizhny Novgorod region, massive losses of roots due to fungi forming sclerotia were observed. At a storage temperature of +1 °C, there was widespread infection of carrot root tissues by mycelium with tissue softening, leading to subsequent decay, severe weight loss, and loss of market quality of roots (Figure 1). In addition, mycelial development was noticed on the wooden parts of storage pallets, through which the mycelium rapidly colonized other batches of roots. The survey showed that these fungi grow rapidly on carrot roots and form sclerotia on the surface of affected organs. The morphology of the pathogen (white floccose mycelium with the formation of dark sclerotia) indicated fungi of the genus *Sclerotinia* [2,3].

In total, 22 strains preliminarily assigned to *Sclerotinia* sp. were isolated from root samples; however, since they were uniform, two strains from carrot differing in growth characteristics on PDA were used in further work (one grew slowly as an olive-grayish mycelium and produced small sclerotia scattered randomly over the plate, the second produced large sclerotia located mainly at the periphery of the plate). Both selected isolates in pure culture on PDA produced light-colored mycelium, on which dark-colored sclerotia subsequently formed; they caused zones of chlorosis and mycelial growth on leaf tissue and root discs around the site of inoculation with an agar plug; they were most similar (>95%) by sequences of *rDNA-ITS* regions to the corresponding sequences of reference strains *S. sclerotiorum* and *S. nivalis* in a BLAST search (https://blast.ncbi.nlm.nih.gov/Blast.cgi, accessed on 21 September 2025). Annotated sequences of *rDNA-ITS* regions of *Sclerotinia* strains were deposited in the NCBI GenBank database. Characteristics of the strains and GenBank *rDNA-ITS* numbers are shown in Table 1.

In addition, the study used a strain of *S. sclerotiorum* isolated from an infected rapeseed stem (SP1) at the end of the 2022 growing season, and a strain of *S. sclerotiorum* from soybean (SC382), detected as sclerotia in a batch of uncleaned soybean grain produced in 2024. Preliminary pathogenicity testing of these strains showed that they were also capable of infecting rapeseed and soybean, respectively. Thus, further work was carried out using 4 strains described in Table 1.

### 2.2. Phylogeny and Taxonomy

#### 2.2.1. General Genomic Features of *S. nivalis* SM8

The draft genome of *S. nivalis* SM8 (Supplementary File S1) provides initial evidence of its species identity. The genome size (~48 Mb) and GC content (~39.8%) of SM8 are similar to those reported for *S. nivalis* SnTB1 (~48 Mb, 39.5% GC), and clearly distinct from the smaller-genome, higher-GC *S. sclerotiorum* (~38 Mb, 41.6% GC [24]). This suggests that SM8 belongs to the *S. nivalis* lineage. To confirm this, we employed multilocus and *ITS* phylogenetic analysis in comparison with known *Sclerotinia* species including both full-genome sequences and separate genes sequences.

#### 2.2.2. Multilocus Phylogenetic Analysis

**Figure 2:**
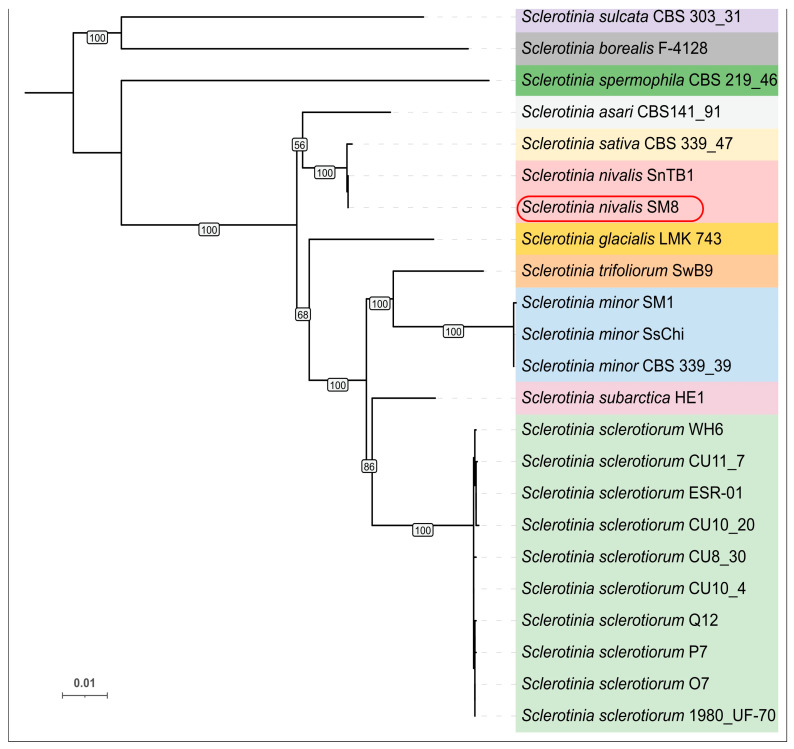
Maximum-likelihood phylogenetic tree of 23 *Sclerotinia* species with sequenced genomes (the list of genomes and GenBank assembly accessions are provided in Supplementary Table S1) based on the concatenated *ITS*, *tubB*, *his3*, *g3pdh*, *hsp60*, and *rpb2* sequences (total alignment ~4.6 kb). Bootstrap support values are indicated on the branches, some bootstrap values for internal short-length branches were removed for clarity. Different clades are colored in different colors, *S. nivalis* strains are colored red and the novel strain SM8 is outlined. The tree is midpoint-rooted; scale bar denotes 0.01 substitutions per site.

The concatenated six-locus phylogeny (the internal transcribed spacer (*ITS*) region and five protein-coding genes—β-tubulin (*tubB*), histone H3 (*his3*), glyceraldehyde-3-phosphate dehydrogenase (*g3pdh*), heat shock protein 60 (*hsp60*), and RNA polymerase II second-largest subunit (*rpb2*)) used genes obtained from 23 sequences genomes. The resultant tree resolved *Sclerotinia* species into well-supported clades (Figure 2). SM8 grouped unequivocally with the *S. nivalis* reference strain SnTB1, with zero nucleotide differences across the concatenated loci. *S. sativa* (CBS 339.47), which was originally described from ginseng, clustered as a very close sister to the *S. nivalis* clade. In our ML tree, SM8 and SnTB1 formed a clade together with *S. sativa*, distinct from other species. The branch separating *S. nivalis* (incl. SM8) and *S. sativa* was very short, reflecting minimal sequence divergence. In fact, *S. sativa* differed from *S. nivalis* by only a few SNPs in *g3pdh* and *ITS*. Other *Sclerotinia* species formed separate clades: for example, *S. sclerotiorum* (strain 1980 UF-70 and others) clustered with its close relative *S. trifoliorum*, and *S. minor* formed its own clade. The *S. nivalis*/*S. sativa* group was monophyletic and apart from those clusters. Overall, the MLST tree indicates that strain SM8 is genetically conspecific with *S. nivalis*, and it also highlights the remarkably short genetic distance between *S. nivalis* and *S. sativa* (see Discussion).

#### 2.2.3. *ITS* Phylogeny

**Figure 3:**
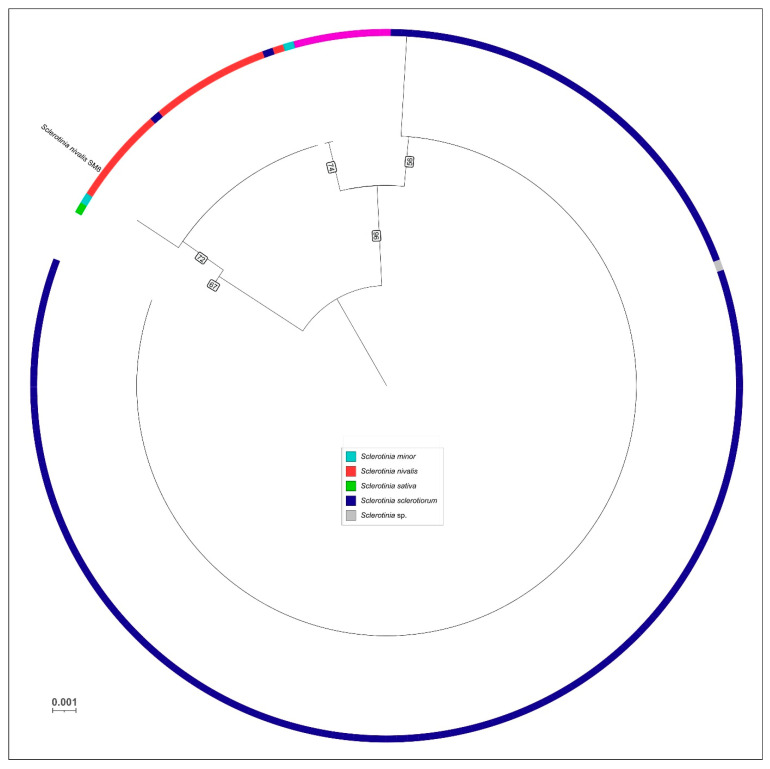
Circular ML phylogenetic tree of *Sclerotinia* based on *ITS* sequences from 200 global isolates. Species clades are color-coded according to legend and based on NCBI annotations. Strain SM8 falls within the *S. nivalis* clade, grouping with other *S. nivalis* isolates from diverse geographic origins. The tree is midpoint-rooted. Bootstrap support values are indicated on the branches, some bootstrap values for internal short-length branches were removed for clarity. Scale bar denotes 0.001 substitutions per site.

The *ITS* region alone was also analyzed to compare SM8 with a broad range of *Sclerotinia* isolates worldwide. In the *ITS* phylogeny (200 sequences), strain SM8 again clustered within the *S. nivalis* group (Figure 3). All known *S. nivalis ITS* sequences formed a cohesive clade with minimal internal variation, and SM8 fell into this clade, reinforcing its identity. The overall topology of the *ITS* tree was consistent with the multilocus results, though with lower resolution. Notably, *S. nivalis* (including SM8) did not show overlap with the *ITS* variability of *S. sclerotiorum*/*minor*/*trifoliorum* complexes, underscoring that it is a well-defined lineage by *ITS* as well.

#### 2.2.4. ANI Comparisons

**Figure 4:**
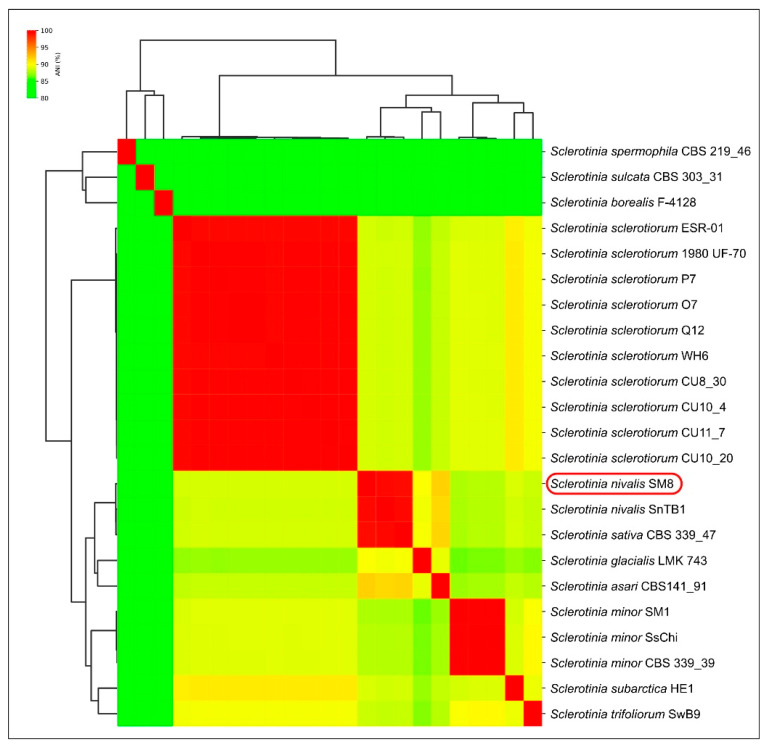
Heatmap of pairwise Average Nucleotide Identity (ANI) percentages among selected *Sclerotinia* genomes, with hierarchical clustering dendrogram (the list of genomes and GenBank assembly accessions are provided in Supplementary Table S1). Each cell shows the ANI value (%) between the genome in that row and column (darker red = higher identity).

To complement the phylogenetic evidence, we compared average nucleotide identities between genomes (Figure 4). The ANI analysis reveals that SM8 is nearly identical to *S. nivalis* SnTB1 at the whole-genome level (ANI = 99.37%). This clearly indicates SM8 and SnTB1 belong to the same genomic species. SM8 also shows very high genome-wide identity to *S. sativa* CBS 339.47 (99.27% ANI), only about 0.1% lower than its identity to SnTB1. In contrast, ANI values between SM8 (or SnTB1) and other *Sclerotinia* species are substantially lower. For example, SM8 vs. *S.* *sclerotiorum* is around ~88–90% ANI, and SM8 vs. *S.* *minor* is in the low 90% range, reflecting the larger evolutionary distance. The clustering dendrogram from the ANI matrix (Figure 4) places SM8 together with SnTB1 and *S. sativa* in a tight cluster (consistent with them being very closely related genomes). That cluster is clearly distinct from the cluster containing *S. sclerotiorum* and *S. trifoliorum* (which share ~90% ANI with each other) and from the group containing *S. minor* and its relatives. These genome-scale data independently confirm that strain SM8 is allied with *S. nivalis* and furthermore suggest that *S. sativa* might be genomically conspecific or nearly so with *S. nivalis* (99.3% ANI). Thus, all phylogenetic and genomic evidence supports the identification of SM8 as *Sclerotinia nivalis*.

### 2.3. Mycelial Growth and Sclerotia Formation In Vitro

**Figure 5:**
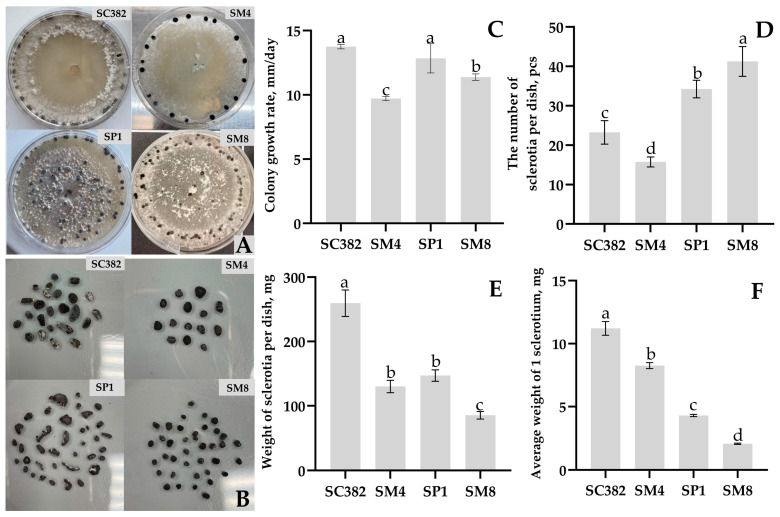
Features of growth and development of *Sclerotinia* strains: appearance and characteristics of sclerotia arrangement (**A**); shape and number of sclerotia obtained from one plate (**B**); colony growth rate (**C**), number of sclerotia from one plate (**D**), mass of sclerotia from one plate (**E**) and average mass of one sclerotium (**F**) when cultured on PDA medium. *Sclerotinia sclerotiorum*—strains SC382, SP1, SM4, *Sclerotinia nivalis*—strain SM8. Different letters indicate a significant difference in values, according to Duncan’s test, at *p* = 0.05. All tests were carried out four times. The standard deviation (SD) is shown for each bar.

When cultivated on PDA, all isolated strains grew successfully and formed sclerotia, but significant differences were noted in growth rate and colony morphology. *S. sclerotiorum* isolates showed rapid radial growth with dense, abundant white mycelial mats, often with pronounced concentric zoning (Figure 5A). Hyphae were septate, hyaline, 4–8 µm thick, with abundant aerial mycelium. In mature cultures, typical sclerotia formed-rounded or irregular black compact structures with a white core, 1.5 to 5 mm in size, located both on the surface and inside the agar layer. The *S. nivalis* strain SM8 exhibited relatively slow growth and formed loose, less compact colonies with weakly developed aerial mycelium. Hyphae were thinner (3–6 µm), septate, and hyaline, with a diffuse colony margin. Sclerotia in *S. nivalis* were unevenly distributed over the medium and were smaller in size (1–3 mm) (Figure 5B).

Radial mycelial growth was observed already 24 h after inoculation of agar plugs with mycelium onto the medium. In terms of radial growth rate expressed in mm/day, the highest values were shown by *S. sclerotiorum* SC382 (13.7 ± 0.2 mm/day), followed by *S. sclerotiorum* SP1 (12.9 ± 1.1 mm/day), *S. nivalis* SM8 (11.4 ± 0.25 mm/day), and finally *S. sclerotiorum* SM4 (9.7 ± 0.2 mm/day) (Figure 5C). Thus, in terms of growth rate on PDA, the strains *S. sclerotiorum* SC382 and SP1 can be considered the fastest growing, whereas the other strains exhibited slowed growth.

All strains formed dark sclerotia on PDA within 7–10 days of cultivation, with substantial differences among strains in morphology and number of sclerotia. In *S. sclerotiorum* SC382 and SM4, sclerotia began to form at the colony periphery after the mycelium had covered the entire surface of the plate, whereas in *S. sclerotiorum* SP1 and *S. nivalis* SM8, sclerotia appeared even before the mycelium closed in the center of the plate. On day 9 of cultivation, it was noted that in *S. nivalis* SM8, sclerotia were small, numerous, and distributed in concentric rings across the colony, giving the central zone of the mycelium an olive-gray tint. In contrast, in *S. sclerotiorum* strains, sclerotia were large, irregularly shaped, and located mainly at the edge of the plate, while the mycelium remained white and dense (Figure 5A).

The number of sclerotia varied significantly, from 15.7 ± 1.25 sclerotia per plate in *S. sclerotiorum* SM4 to 41.2 ± 3.7 in *S. nivalis* SM8 (Figure 5D). *S. sclerotiorum* SP1 also formed a relatively large number of sclerotia (34.2 ± 2.2 per plate), and the strain *S. sclerotiorum* SC382 produced on average 23.2 ± 2.9 per plate. Sclerotia mass correlated with their number: the total weight of sclerotia per plate ranged from 85.2 ± 5.9 mg in *S. nivalis* SM8 to 259.5 ± 20.6 mg in *S. sclerotiorum* SC382 (Figure 5E). On average, a single sclerotium of *S. nivalis* SM8 weighed only 2.1 ± 0.04 mg, whereas in *S. sclerotiorum* SC382, the average mass of one sclerotium reached 11.2 ± 0.54 mg (Figure 5F). Thus, *S. sclerotiorum* strains produced larger sclerotia in smaller numbers, whereas the *S. nivalis* strain produced small sclerotia in large numbers. Statistical analysis confirmed significant differences among all strains in radial growth rate, number, and mean mass of sclerotia. Detailed statistical parameters are provided in Supplementary Table S2.

### 2.4. Effect of Cultivation Temperature on Growth and Development of S. sclerotiorum and S. nivalis In Vitro

**Figure 6:**
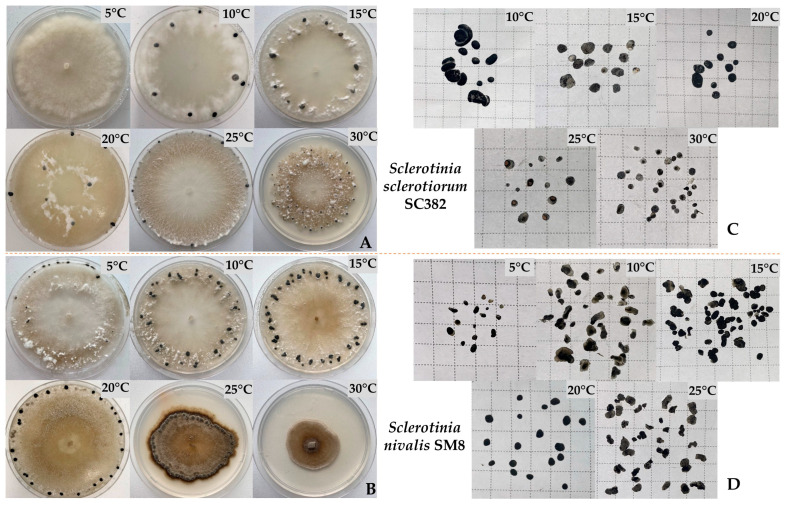
Appearance of cultures, characteristics of sclerotia arrangement, and number of sclerotia of *S. sclerotiorum* strain SC382 (**A**,**C**) and *S. nivalis* strain SM8 (**B**,**D**) obtained from a single plate on PDA medium at temperatures of 5–30 °C.

For an in-depth comparison of the species, *S. sclerotiorum* SC382 and *S. nivalis* SM8 were selected as the most typical for each species. The strains were cultivated at different temperatures (5–30 °C), and differences were revealed in colony morphology, growth rate, and features of sclerotia formation (Figure 6A–D).

**Figure 7:**
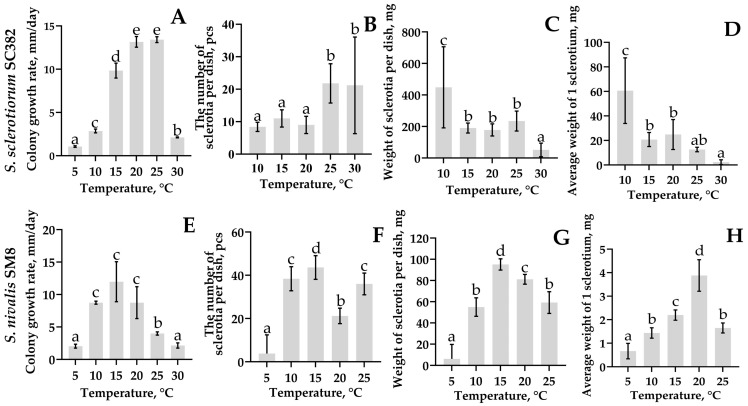
Colony growth rate (**A**,**E**), number of sclerotia per plate (**B**,**F**), mass of sclerotia per plate (**C**,**G**), and average mass per sclerotium (**D**,**H**) during cultivation of *Sclerotinia sclerotiorum* strain SC382 and *Sclerotinia nivalis* strain SM8, respectively, on PDA medium at various temperatures. Different letters indicate a significant difference in values, according to Duncan’s test, at *p* = 0.05. All tests were carried out five times. The standard deviation (SD) is shown for each bar.

*Sclerotinia sclerotiorum*: The SC382 strain showed intensive radial growth and formed sclerotia in the temperature range 15–25 °C (Figure 7A). The highest radial growth rate was recorded at 25 °C and was 13.4 ± 0.3 mm/day. The strain covered the entire surface of the medium in a Petri dish by day 3 and formed 21.8 ± 4.8 sclerotia per plate (Figure 7B).

Under cultivation at 10 °C, the number of sclerotia was lower (8.4 ± 1.4 pcs.), but their total mass reached maximum values (up to 448.4 ± 207.4 mg); accordingly, the formation of large sclerotia was observed (Figure 6A and Figure 7C,D). At 15 °C, the radial growth rate of the strain decreased to 9.8 ± 0.7 mm/day, but this led to the formation of numerous and intensely melanized structures. At 30 °C, colonies developed slowly (radial growth rate 2.12 ± 0.04 mm/day), the number of sclerotia remained high (21.2 ± 11.8 pcs.), but sclerotia mass dropped to minimal values (51.6 ± 34.6 mg). Under cultivation at 5 °C, radial growth slowed to 1.06 ± 0.07 mm/day and no sclerotiogenesis was observed (Figure 6A).

*Sclerotinia nivalis*: The SM8 strain developed actively in the temperature range 10–20 °C (Figure 7B). The optimum for cultivation was 15 °C, at which the radial growth rate reached 11.9 ± 2.5 mm/day, the colony covered the entire plate on average by day 7 of cultivation with the formation of 43.6 ± 4.4 sclerotia per plate with a total mass of 95.2 ± 4.3 mg and an average sclerotium mass of about 2.2 ± 0.2 mg (Figure 7E–H). At 10 °C, the radial growth rate slowed to 8.7 ± 0.2 mm/day, the strain covered the entire plate by day 10, and the number of sclerotia was 38.4 ± 4.5 (with an average mass of 54.9 ± 7.0 mg). At 20 °C, the number of sclerotia was lower (21.2 ± 2.8), but the mass of one sclerotium remained high (3.8 ± 0.54 mg), indicating the formation of fewer but larger structures. At 25 °C, sclerotia productivity decreased and was 36.0 ± 4.06 pcs. with an average mass of 59.2 ± 8.2 mg. At 5 °C, the mycelium grew extremely slowly (2.0 ± 0.24 mm/day), sclerotia formed only in two of five replicates (3.8 ± 6.9 pcs.; with an average mass per plate of 6.1 ± 10.9 mg), and at 30 °C development ceased and sclerotia were not formed at all.

Thus, *S. nivalis* SM8 showed maximum productivity at 15 °C and retained the ability to form sclerotia even at 10 °C, whereas for *S. sclerotiorum* SC382, the optimal temperature was 25 °C. At 10 °C, it formed fewer but larger sclerotia, whereas at 30 °C it maintained limited development, although sclerotia mass decreased sharply.

### 2.5. Assessment of Aggressiveness of Sclerotinia Strains Isolated from Rapeseed, Soybean, and Carrot on Carrot Leaves and Roots

**Figure 8:**
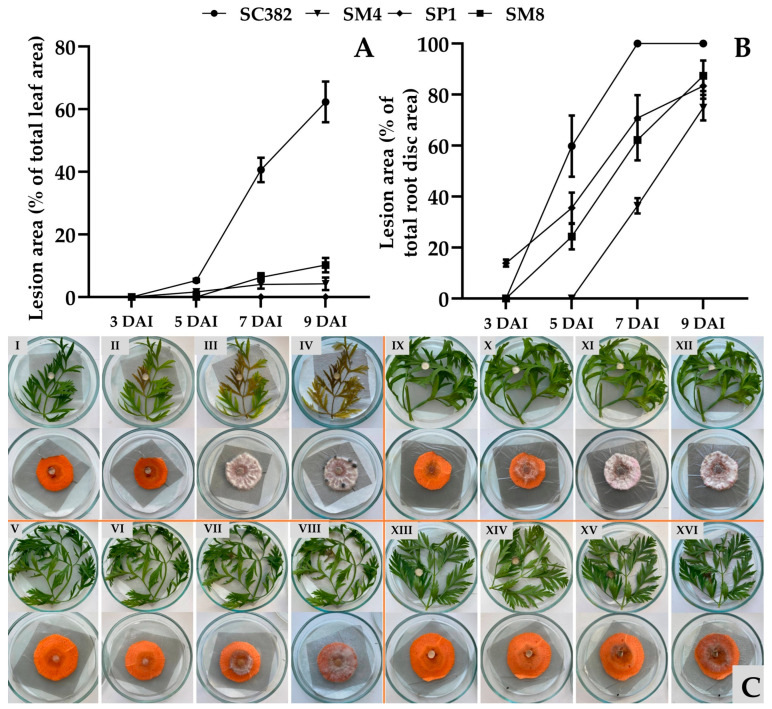
Aggressiveness of *Sclerotinia* strains on carrot root discs and leaves during artificial inoculation. Lesion area on detached carrot leaves (**A**) and root discs (**B**) at 3, 5, 7, and 9 days after inoculation (DAI), and general view of inoculated samples (**C**). I, II, III, IV—*Sclerotinia sclerotiorum* strain SC382; V, VI, VII, VIII—*Sclerotinia sclerotiorum* strain SP1; IX, X, XI, XII—*Sclerotinia sclerotiorum* strain SM4; XIII, XIV, XV, XVI—*Sclerotinia nivalis* strain SM8 at 3, 5, 7, and 9 days after inoculation, respectively. All tests were carried out five times. The standard deviation (SD) is shown for each bar.

All studied strains (except SP1 on leaves) caused the development of necrotic lesions on detached carrot leaves and root discs, but their aggressiveness varied significantly (Figure 8C). The first symptoms appeared 3–5 days after inoculation as local necrotic spots on leaves, and as darkening and water-soaking of tissues on roots (Figure 8C).

Quantitative accounting of the affected area showed clear differences between isolates and tissues (Figure 8C). Upon inoculation of detached leaves, the most aggressive was *S. sclerotiorum* SC382; by day 9 of incubation, 62% of the leaf area was necrotized. The *S. sclerotiorum* SP1 strain from rapeseed was non-pathogenic on carrot leaves, while *S. sclerotiorum* SM4 and *S. nivalis* SM8 occupied an intermediate position with leaf lesion areas of 4.2 and 10.2%, respectively, on day 9 after inoculation (Figure 8A).

On root discs, the dynamics were different (Figure 8B). Already on the third day after inoculation, the *S. sclerotiorum* SP1 strain formed necrotic foci on 14% of the disc area, whereas in discs inoculated with other strains, no visible symptoms were observed at this exposure. The maximum differences between strains appeared by day 9. Thus, the necrotic area in *S. sclerotiorum* SC382 reached 100%, followed by *S. nivalis* SM8 (87%) and *S. sclerotiorum* SP1 and SM4 (83 and 75%, respectively).

Thus, the *S. sclerotiorum* SC382 strain from soybean proved to be the most aggressive both on leaves and on root discs of carrot. The *S. nivalis* SM8 strain did not show strong aggressiveness on leaves; however, together with other strains, it actively infected root discs.

### 2.6. Sensitivity of Strains to Boscalid, Fluazinam, and Pyraclostrobin Using EC_50_

Assessment of the sensitivity of *S. sclerotiorum* and *S. nivalis* strains to boscalid, fluazinam, and pyraclostrobin showed statistically significant differences between them (Duncan’s test, *p* = 0.05; Table 2, Figure S1).

For boscalid, EC_50_ values ranged from 0.1057 ± 0.0063 to 0.2017 ± 0.0069 µg/mL. The lowest value was recorded for *S. sclerotiorum* SM4, and the highest for *S. sclerotiorum* SC382. The *S. nivalis* SM8 strain was characterized by an intermediate EC_50_ value (0.1908 ± 0.0015 µg/mL). For fluazinam, the range of EC_50_ values was from 0.0024 ± 0.0001 to 0.0107 ± 0.0007 µg/mL. Similar values were noted for *S. sclerotiorum* SC382 and SM4 (0.0024 ± 0.0001 and 0.0024 ± 0.0003 µg/mL, respectively). A higher EC_50_ value was observed in *S. nivalis* SM8 (0.0107 ± 0.0007 µg/mL). For pyraclostrobin, EC_50_ values ranged from 0.0908 ± 0.002 to 0.1317 ± 0.0069 µg/mL. The minimum value was noted in *S. sclerotiorum* SM4, and the maximum in *S. sclerotiorum* SC382. The EC_50_ value for *S. nivalis* SM8 was 0.1175 ± 0.002 µg/mL.

## 3. Discussion

The present study provides new evidence that *Sclerotinia nivalis*, a species mainly associated with alpine and boreal habitats, can develop at low temperatures and pose a latent risk during carrot storage. Unlike common storage pathogens, *S. nivalis* demonstrates active growth at 0–5 °C, which may allow the fungus to remain undetected on asymptomatic roots while gradually producing mycelium or sclerotia over extended storage periods. This cold-tolerant behavior highlights the importance of characterizing its pathogenic and physiological traits, especially in regions where long-term storage under refrigerated conditions is essential for supply chain continuity. To the best of our knowledge, the present study reports for the first time the molecular-genetic identification of *S. nivalis* as a pathogen of carrot in Russia, expanding the geography of the distribution of this species worldwide. Multiple lines of evidence identify SM8 as *S. nivalis*. In the six-locus ML phylogeny, SM8 groups with the *S. nivalis* reference SnTB1 with no detectable sequence divergence. In the global ITS dataset, SM8 falls within the *S. nivalis* cluster, distinct from other *Sclerotinia* species. Whole-genome ANI between SM8 and SnTB1 is 99.37%, indicating near-identity and supporting conspecific status. Obtained data expand the documented diversity of *S. nivalis* by adding a new isolate from carrot.

Genomic analysis indicated a very close relatedness of *S. nivalis* and *S. sativa*. *Sclerotinia sativa* [25] has been treated as a separate species. However, *S. sativa* CBS 339.47 shows a very small genetic distance to *S. nivalis*. In the six-gene tree it is sister to *S. nivalis* with negligible branch length; only a few SNPs are observed in specific loci (*ITS* and *g3pdh*). Genome-wide identity is similarly high: ANI between *S. sativa* (CBS 339.47) and *S. nivalis* (SM8/SnTB1) is ~99.3%, a level typical of intraspecific comparisons. Despite high ANI values, the species differ in sclerotial morphology and ecological adaptation to low-temperature niches, which may justify maintaining them as separate taxa. A previous analysis based on *ITS* sequences has placed *S. sativa* closer to *S. minor* [24], but multilocus and genomic data indicate a closer relationship to *S. nivalis*. A formal synonymy is not proposed here; evaluation of original descriptions and any consistent phenotypic differences (e.g., sclerotial size, host range) is warranted. Nevertheless, if additional *S. sativa* isolates exhibit < 0.5% genomic divergence from *S. nivalis*, conspecific status should be considered.

ANI is widely used in bacteriology (95–96% as a common species boundary), but universal thresholds are not established for fungi [26,27,28]. High ANI can occur between recently diverged or slowly evolving fungal species. The >99% ANI observed between *S. nivalis* and *S. sativa* suggests potential conspecificity, pending further phenotypic and population-level evidence. Notably, ANI contrasts among recognized species (e.g., *S. sclerotiorum* vs. *S.* *nivalis* at ~88–90%) reveal clear genomic discontinuities consistent with species boundaries.

This study is based on a single *S. nivalis* strain, which confirms the presence of the species but does not allow evaluation of its population diversity or geographic distribution. Therefore, the term «first report» refers specifically to the first molecular identification rather than an assessment of prevalence. Previously, this species was noted only on ornamental and wild plants in Russia [22,23]. Globally, *S. nivalis* is known as the causal agent of white mold and snow mold in temperate and cold climates [9,20,21]. On carrot, it was first described in Japan [9], but subsequent reports of *S. nivalis* infection on this crop are virtually absent. Our *S. nivalis* strain SM8 shares key features with Japanese and Chinese *S. nivalis* strains, including small sclerotia and optimal growth at 10–15 °C, consistent with their adaptation to cool temperate climates [9,13,14].

In this regard, our study is the second in the world to prove the pathogenesis of *S. nivalis* on carrot. It is likely that *S. nivalis* is inferior to *S. sclerotiorum* in competitiveness, manifesting only at low storage temperatures. Our data show that long-term storage of carrot at +1 °C creates conditions for its active development, whereas under these conditions *S. sclerotiorum* is apparently under stress and acts only as a component of the pathocomplex [2]. The ability of *S. nivalis* to grow and form sclerotia at 0–5 °C [12] is confirmed in our experiments. It is likely that this species was present earlier but was misidentified as *S. minor* or *S. sclerotiorum* due to similarity of symptoms [13].

Morphologically, *S. nivalis* differs from *S. sclerotiorum* by slow growth and the formation of small sclerotia (2–5 mm) in larger numbers [9,13]. In *S. sclerotiorum*, sclerotia are larger (5–15 mm) and are located mainly at the colony periphery. These differences reflect different survival strategies: *S. nivalis* forms numerous small sclerotia to survive cold, whereas *S. sclerotiorum* forms larger, more resource-rich structures [12]. The optimal growth temperature for *S. nivalis* was 15 °C, and for *S. sclerotiorum* 20–25 °C, which is consistent with published studies and the ecological niches of the pathogen [18,21]. Thus, *S. nivalis* is adapted to development under cool storage conditions, where competition from other phytopathogens is minimal. The ability of *S. nivalis* to grow and sporulate at low temperatures highlights its potential risk for carrot storage facilities and northern agricultural regions.

Comparison of strain aggressiveness showed that *Sclerotinia* strains isolated from different hosts are capable of infecting carrot with varying degrees of aggressiveness. The most aggressive was the *S. sclerotiorum* SC382 strain from soybean, which caused necrosis of up to 62% of the leaf area and complete decay of root discs within 9 days. The strain from rapeseed (*S. sclerotiorum* SP1) infected only roots, indicating possible tissue specialization [8]. The results obtained demonstrate the risk of cross-infection when rotating carrot with soybean or rapeseed, as previously noted in Canada [7]. This underscores the need to consider sclerotiniosis when designing crop rotations and laying carrots in storage.

For the first time, the sensitivity of *S. nivalis* to fungicides was evaluated. The *S. nivalis* SM8 strain showed sensitivity comparable to *S. sclerotiorum* to boscalid and pyraclostrobin [29], but four times lower sensitivity to fluazinam. This may explain cases of weak fluazinam efficacy in cold storage facilities where *S. nivalis* predominates and dictates the need for further studies to assess population sensitivity of *S. nivalis* to fungicides. Fluazinam is a broad-spectrum fungicide whose primary mode of action is uncoupling oxidative phosphorylation in mitochondrial membranes, leading to disruption of ATP synthesis and induction of oxidative stress. Several studies demonstrated that fluazinam exposure triggers a strong glutathione-dependent detoxification response in *Sclerotinia*, including upregulation of glutathione S-transferases, increased GSH/GSSG turnover, and activation of antioxidant enzymes [30,31]. Such responses may partly explain the higher EC_50_ observed in SM8, as isolates capable of faster neutralization of reactive intermediates or enhanced antioxidant buffering can display reduced in vitro sensitivity to fluazinam. Given the variability in fluazinam EC_50_ values, routine sensitivity monitoring and rotation with fungicides of different FRAC groups are recommended. Boscalid and pyraclostrobin maintained high activity against both species, making them preferable in integrated systems for protecting carrot from white mold.

## 4. Materials and Methods

### 4.1. Isolation of Sclerotinia Strains

During the inspection of vegetable storage facilities, softened carrot tissues with dark-colored sclerotia on white mycelium were collected, placed in paper bags, transported to the laboratory, and stored at 4 °C until analysis. Pure cultures were obtained following the methods of [32,33] with modifications. Dense, mature, dark sclerotia at least 1 mm in diameter were extracted from infected plant tissues showing typical symptoms of white mold using a sterile needle and placed in 1.5 mL Eppendorf tubes. The sclerotia were washed three times with water by pipetting to remove plant tissue and soil particles and subsequently treated with 70% ethanol for 30 s, 1% sodium hypochlorite solution for 2 min, and rinsed three times with sterile distilled water to remove residual disinfectants.

Surface-sterilized sclerotia were cut with a sterile scalpel and forceps in a laminar flow cabinet. The cut sclerotia were transferred to Petri dishes containing potato dextrose agar (PDA) (g/L: potato broth from 300 g potatoes—300.0 mL; glucose—20.0; agar—17.0; water to 1 L) supplemented with antibiotics (streptomycin sulfate and chloramphenicol, (Central Drug House (P) Ltd., Delhi, India), each at 100 mg/L) to suppress bacterial contamination. The pH values were adjusted to 5.6 by measuring using a SanXin PHS-3D-01 pH meter (SanXin Instrumentation, Shanghai, China) prior to autoclaving. Plates were incubated at 20 ± 1 °C in an KB 23 incubator (BINDER GmbH, Tuttlingen, Germany), and white mycelium growth was observed after 2–3 days, followed by sclerotia formation on days 7–9. A hyphal fragment from the colony margin not in contact with the original sclerotium was cut with a sterile needle and transferred to fresh PDA. This subculturing step was repeated three times to obtain a pure culture. Pure isolates were maintained on agar at +4 °C and in 20% glycerol at −80 °C. Strains isolated from rapeseed and soybean were purified and stored in the same way.

### 4.2. Pathogenicity Testing of Strains on Host Plants

Pathogenicity tests were conducted according to [34] with some modifications. Soybean cv. Kasatka, winter rapeseed cv. Garant, and carrot cv. Shantane 2461 were grown in a glass greenhouse at 28/22 °C (14 h day/10 h night) under natural light and watered as needed. Plants were cultivated in peat-perlite substrate (Veltorf, Vologda, Russia) in plastic pots (0.5 L cell volume, AgrofloRaPak, Vologda, Russia) until 3–4 true leaves appeared. Three leaves of each plant species were used for inoculation. For inoculation, 7 mm mycelium plugs (approximately 15–20 mg of fresh biomass) were cut from 2-day PDA cultures of each isolate using a sterile cork drill, they were placed in the center of each sheet and gently pressed against the surface. Each isolate was used to inoculate the host species from which it had originally been obtained. Growth conditions remained unchanged until the end of the experiment. On day 5 after inoculation, the presence of chlorosis zones and mycelial growth on inoculated leaves was recorded. Isolates that did not cause symptoms were excluded from further work. Mock-inoculated controls (agar plugs without mycelium) remained symptom-free throughout the experiment.

After the experiment, Koch’s postulates were verified by re-isolating fungi from infected tissues on PDA with antibiotics and purifying to pure culture, as described previously. The identity of re-isolated strains was confirmed by comparing their morphological characteristics with those of the original isolates used for inoculation, as well as by sequencing the rDNA-ITS regions and comparing nucleotide sequences.

### 4.3. Identification of Sclerotinia Strains

Preliminary identification was carried out based on colony morphology, presence, arrangement, and size of sclerotia on PDA according to [9]. Mycelium morphology was examined under an Axiolab 5 microscope (Carl Zeiss AG, Oberkochen, Germany).

For final identification, DNA was extracted from 7-day-old mycelium using the «Phytosorb» DNA extraction kit (Syntol LLC, Moscow, Russia) according to the manufacturer’s protocol. Reaction mixtures contained 5 µL of 5× Master-mix (5× MasDDTaqMIX-2025, Dialat LTD, Moscow, Russia), 10 µM of each primer (ITS4 (5′-TCCTCCGCTTATTGATATGC-3′) and ITS5 (5′-GGAAGTAAAAGTCGTAACAAGG-3′)), 5 ng of target DNA, and PCR-grade water (Syntol LLC, Moscow, Russia) up to a total volume of 25 µL. PCR amplification of *rDNA-ITS* regions was performed in a T100 thermal cycler (Bio-Rad, Hercules, CA, USA) according to [35]. Amplicons were separated by electrophoresis in 1.5% agarose gel, stained with ethidium bromide in 0.5× TBE buffer, and visualized using a Gel Doc XR+ system (Bio-Rad, Hercules, CA, USA). PCR fragments were excised and purified using the ColGen kit (Syntol LLC, Moscow, Russia) according to the manufacturer’s instructions. Sequencing of purified PCR products was performed by the Sanger method using the BigDye Terminator v3.1 Cycle Sequencing Kit (Life Technologies ThermoFisher, Waltham, MA, USA) and an automatic DNA analyzer 3730 (Thermo Fisher Scientific, Waltham, MA, USA) at Syntol LLC. The obtained sequences were compared with the GenBank database using the BLASTn algorithm. A species was considered reliably identified when sequence similarity with the type strain was ≥95% [36].

### 4.4. Genome Sequencing and Assembly

The whole genome of *S. nivalis* strain SM8 was sequenced and assembled into a draft genome. Genomic DNA was extracted from lyophilized mycelium by phenol–chloroform purification and sheared with a Bioruptor sonicator (Diagenode, Liège, Belgium). Samples underwent quality control by agarose gel electrophoresis (AGE). Quality control was considered passed if the sample on the electrophoregram was at the same level or higher than the 10,000 bp marker. Genomic DNA concentrations were measured on a Qubit 3.0 fluorometer (Life Technologies ThermoFisher, Waltham, MA, USA) using the dsDNA BR Assay Kit (Life Technologies ThermoFisher, Waltham, MA, USA) according to the manufacturer’s protocol. Libraries were prepared from genomic DNA using the MGIEasy FS DNA Library Prep Set according to the manufacturer’s protocol (MGI Tech Co., Shenzhen, China). A total of 260 ng of genomic DNA was used for library preparation. After fragmentation, size selection was performed using MGIEasy DNA Clean Beads—0.5× to remove long fragments and 0.36× of the initial volume to remove short fragments. Library quality control was performed by agarose gel electrophoresis (AGE). Library concentrations were measured on a Qubit 3.0 fluorometer (Life Technologies Thermo Fisher, Waltham, MA, USA) using the dsDNA HS Assay Kit (Life Technologies Thermo Fisher, Waltham, MA, USA) according to the manufacturer’s protocol. The finished library was circularized and sequenced in paired-end mode on the DNBSEQ-G99 platform using the Universal Sequencing Reaction Kit G99 SMApp-DPE150 according to the manufacturer’s protocol (MGI Tech Co., Shenzhen, China). DNB concentrations were measured on a Qubit 3.0 fluorometer (Life Technologies Thermo Fisher, USA) using the ssDNA Kit according to the manufacturer’s protocol. Primary quality control of the sequenced library was performed using FastQC v0.12.1 [37] and MultiQC v1.18 [38]. Subsequently, based on the obtained reports, trimming of fq.gz files and their subsequent assembly with SPAdes v4.0.0 were performed. Assembly quality control was conducted using Kraken2 v2.1.1 [39] (taxonomic diversity, representation of contaminating groups), MetaBAT2 v2.18 [40] (binning, detection of non-target organisms), and QUAST v5.3.0 [41] (general assembly quality assessment). Additionally, contigs associated with bacterial contamination were removed (‘extract_kraken_reads.py’, KrakenTools software [42]). According to the QUAST v5.3.0 report, the N50 is 31,512, the number of contigs is 19,225, of which 5071 are ≥500 bp. The maximum contig length is 255,834, and the GC content is 39.75%. Contigs were inspected and curated in Geneious Prime 2025.0.3 (Biomatters, Inc., Auckland, New Zealand). The draft genome of *S. nivalis* SM8 was deposited in the NCBI GenBank database under the BioProject accession number PRJNA1344670. The size of the SM8 assembly was 47,605,970 bp and the coverage 129.4.

### 4.5. Phylogenetic Analysis and Average Nucleotide Identity Calculations

To determine the phylogenetic placement of strain SM8, multilocus sequence typing (MLST) using six loci (the internal transcribed spacer (*ITS*) region and five protein-coding genes—β-tubulin (*tubB*), histone H3 (*his3*), glyceraldehyde-3-phosphate dehydrogenase (*g3pdh*), heat shock protein 60 (*hsp60*), and RNA polymerase II second-largest subunit (*rpb2*)) was conducted. Sequences from SM8 were aligned with reference sequences of multiple *Sclerotinia* species (including *S. nivalis* SnTB1, *S. sativa* CBS 339.47, *S. sclerotiorum*, *S. minor*, *S. trifoliorum*, and others) using the MAFFT v7.490 [43] applying the L-INS-i iterative refinement algorithm, and other settings were default. Phylogenetic trees were inferred under maximum likelihood in IQ-TREE 2 [44]. The commands ‘-m TEST’ and ‘-bb 1000’ were used to automatically select the best-fit substitution model and assess branch support with 1000 bootstrap replicates. The resultant phylogeny was visualized using iTOL v7 [45]; the tree was midpoint-rooted and annotated with species labels and bootstrap support values.

The phylogenetic tree based on *ITS* sequences was obtained using a wide collection of *Sclerotinia* isolates worldwide. Approximately ~200 *ITS* sequences were retrieved from GenBank, representing all major *Sclerotinia* species across a global distribution (including multiple isolates of *S. sclerotiorum*, *S. minor*, *S. nivalis*, etc.). These *ITS* sequences were aligned with MAFFT (L-INS-i) and manually inspected. A maximum likelihood *ITS* tree was generated with IQ-TREE 2 under the same parameters as above (automatic model selection, 1000 bootstrap replicates). The resultant *ITS* phylogeny was also visualized in iTOL, with midpoint rooting.

Average Nucleotide Identity (ANI) calculations were obtained using a panel of 23 publicly available *Sclerotinia* genomes. This dataset included the genomes of *S. nivalis* SnTB1, *S. sativa* CBS 339.47, multiple strains of *S. sclerotiorum*, and representatives of *S. minor* and *S. trifoliorum*, among others. ANI was computed using FastANI v1.34 [46]. The pairwise ANI results were visualized as a clustered heatmap and dendrogram using ANIclustermap (https://github.com/moshi4/ANIclustermap, accessed 10 September 2025).

### 4.6. Evaluation of Radial Growth Rate and Sclerotia Formation

An agar plug with mycelium obtained as described in Section 4.2 was transferred to the center of a new Petri dish containing PDA, sealed with Parafilm, and incubated in an incubator at 20 ± 0.5 °C. The radial colony growth was measured daily, starting from the second day of cultivation and continuing for nine days, in two perpendicular directions using a digital caliper ADA Mechanic 150 PRO (ADA INSTRUMENTS Co LTD., Shenzhen, China). The average radial growth rate of the mycelium was calculated according to [2] using the formula:

V = D_n_ − D_0_/n, (1)

where V—average growth rate (mm/day); D_n_—colony diameter on day n; D_0_—initial diameter (7 mm); n—number of incubation days.

Sclerotia formation was assessed on the ninth day after the completion of active growth using the same Petri dishes where radial growth was measured. Starting from day 4 of cultivation, plates with cultures were transferred from a dark incubator to an incubator with alternating light conditions (12 h light/12 h dark) at the same temperature (20 ± 0.5 °C) to stimulate sclerotial morphogenesis. All sclerotia formed on the plate were removed with tweezers, counted, dried for 24 h in an incubator at 30 °C to air-dry weight, weighed on analytical balances Quintix (Sartorius AG, Göttingen, Germany), and the average mass of a single sclerotium for each strain was calculated. The experiment was performed in four replications for each strain.

### 4.7. Evaluation of the Effect of Cultivation Temperature on Mycelial Growth and Sclerotia Formation

An agar plug with mycelium obtained as described in Section 4.2 was transferred to the center of a new Petri dish with PDA. Plates were sealed with Parafilm and incubated in hermetic containers to prevent medium desiccation. Cultivation was carried out at six temperature regimes: 5 °C, 10 °C, 15 °C, 20 °C, 25 °C, and 30 °C in a constant-temperature incubator (±0.5 °C) in the dark. Radial mycelial growth was measured on days 3, 7, and 10, and then every 5 days using a digital caliper. The average growth rate was calculated using the formula described in Section 4.6. After complete sclerotia formation, their number was evaluated as described in Section 4.6. The experiment was performed in five replications per strain.

### 4.8. Evaluation of Strain Virulence on Carrot Leaves and Roots

Virulence of strains was assessed according to [34,47] with modifications, using freshly harvested leaves and roots of carrot cv. Shantane 2461 grown as described in Section 4.2. The roots were washed under running water to remove soil particles, sterilized with 70% ethanol, and air-dried in a sterile laminar flow cabinet for 30 min. Leaves were detached with petioles, washed under running water for 10 min, immersed in sterile water for 15 min, and similarly air-dried. Two inoculation variants were used: (I) root discs (slices 5 ± 0.5 mm thick, 30 ± 2 mm in diameter) cut from healthy surface-sterilized carrots using a sterile knife in a laminar cabinet; and (II) carrot leaves of uniform size, cut into fragments 5 ± 1 cm long. Each variant was tested in five replicates (five root discs or five leaves per strain).

Prepared root discs and leaves were placed in sterile glass Petri dishes lined with moistened filter paper (to maintain high humidity) and a layer of sterile foil. Mycelial discs obtained as described in Section 4.2 were gently placed in the center of each root disc or on the upper surface of the leaf (at the mid-petiole region). Dishes were closed with lids, sealed in zip-lock bags, and incubated at 24 °C in the dark. High humidity in the dishes was maintained throughout the experiment by moistening the filter paper daily with sterile water.

Observations were made on days 3, 5, 7, and 9 after inoculation by measuring mycelial growth zones using the LeafDoctor application (https://www.quantitative-plant.org/software/leaf-doctor, accessed 21 June 2025) installed on an iPhone SE 2. Each leaf and root disc was photographed separately and analyzed by adjusting the threshold slider until only symptomatic tissues appeared blue, and the percentage of affected tissue was calculated following the developer’s recommendations [48,49].

### 4.9. Evaluation of Sensitivity to Boscalid, Fluazinam, and Pyraclostrobin Using EC_50_

Sensitivity of the strains to fungicides was assessed according to [29] with modifications. Effective concentrations (EC_50_), at which mycelial growth was inhibited by 50% compared to the control (without fungicide), were determined. Technical-grade active ingredients (a.i. 95%) were used: boscalid (pyridinyl amide, SDHI; manufacturer Haili Guixi Chemical Co. Ltd., Yingtan, China), fluazinam (anilide; Hubei Kang Bao Tai Fine-Chemical Co. Ltd., Hubei, China), and pyraclostrobin (strobilurin, QoI; Hubei Kang Bao Tai Fine-Chemical Co. Ltd., Hubei, China). Before addition to the medium, boscalid and pyraclostrobin were dissolved in 99.8% dimethyl sulfoxide (DMSO; Component-Reaktiv LLC, Moscow, Russia), and fluazinam was dissolved in 99.8% acetone (TD HIMMED LLC, Moscow, Russia) to prepare 1000 mg a.i./mL stock solutions. Serial dilutions were prepared to obtain the required concentrations. Solvent controls (DMSO or acetone) were included at identical final concentrations as in fungicide treatments, and no inhibitory effects on mycelial growth were detected.

The prepared solutions were added with automatic pipettes to sterile glass jars containing previously autoclaved PDA medium cooled to 47 °C in a water bath, gently mixed to avoid bubbles, and poured into sterile Petri dishes (20 mL per plate) using an electric pipetting dispenser Levo Plus (DLAB Scientific Co., Ltd., Beijing, China) in a sterile laminar cabinet. The final concentrations in PDA were: for boscalid: 0 (PDA + DMSO), 0.03, 0.1, 0.3, 1.0, and 5 µg/mL; pyraclostrobin: 0 (PDA + DMSO), 0.025, 0.05, 0.1, 0.2, and 0.4 µg/mL; fluazinam: 0 (PDA + acetone), 0.0025, 0.005, 0.01, 0.05, and 0.1 µg/mL [29,50].

Plates were left for 30 min in the working laminar cabinet for solidification. Agar plugs (7 mm) were cut from the periphery of a 2-day-old colony of each isolate using a laboratory cork borer and placed in the center of each plate with PDA containing the respective fungicide concentration using sterile metal tweezers.

Colony diameters were measured as described in Section 4.6 on the third day of incubation at 20 °C in the dark. Each treatment was performed in triplicate. EC_50_ values were calculated by fitting a logarithmic model to the relationship between colony diameter and fungicide concentration using GraphPad Prism 9.2.0 (GraphPad Software Inc., Boston, MA, USA).

### 4.10. Statistical Analysis and Visualization

All statistical analyses were performed in Statistica 12.0 (TIBCO Software Inc., Palo Alto, CA, USA). Data were first tested for normality using the Shapiro–Wilk test and for homogeneity of variances using Levene’s test. When assumptions were met, differences among isolates were assessed using one-way ANOVA, followed by Duncan’s multiple range test at *p* = 0.05 to separate means. For features of strains growth, pathogenicity assays and fungicides EC_50_ values, each isolate had 3–5 biological replicates as indicated in Supplementary Table S2. Graphs were visualized in GraphPad Prism 9.2.0.

## 5. Conclusions

In the present study, the presence of *S. nivalis* as a causal agent of white mold of carrot (*Daucus carota*) during storage was molecularly confirmed in Russia for the first time, expanding understanding of the species composition of white mold pathogens of this crop. It was established that isolates obtained from soybean and rapeseed are capable of infecting carrot and causing significant damage, with the soybean-derived strain showing the highest aggressiveness on carrot tissues. It was also revealed that *S. nivalis* has a lower temperature optimum (15 °C) and forms a large number of small sclerotia, in contrast to *S. sclerotiorum* (25 °C), which forms fewer but larger sclerotia. For the first time, it was shown that the *S. nivalis* strain is less sensitive to fluazinam compared to *S. sclerotiorum*, which requires attention when choosing fungicides for carrot treatments. The data obtained emphasize the need to monitor the species composition of pathogens, adjust crop rotations with soybean and rapeseed, and implement storage practices for carrot that take into account cross-infection and the ability of cold-tolerant pathogens to affect roots during storage.

## Figures and Tables

**Table 1:** List of *Sclerotinia* strains used in the study.

Name of the Strain	Source (Plant Species)	Growing Region	Year of Isolation	Sclerotinia Species	Genbank rDNA-ITS No.
SC382	Soybean (*Glycine max*)	Orel region, Mtsensk district, Russia	2024	*sclerotiorum*	PV973875
SP1	Rapeseed (*Brassica napus*)	Kaluga Region, Russia	2022		PV973876
SM4	Carrot (*Daucus carota* subsp. *sativus*)	Nizhny Novgorod region, Arzamas district, Russia	2024		PV973879
SM8			2023	*nivalis*	PV973881

**Table 2:** Sensitivity of *Sclerotinia sclerotiorum* and *Sclerotinia nivalis* strains to boscalid, fluazinam and pyraclostrobin.

Strain	Mean Value EC_50_ ± SD (µg/mL)		
	Boscalid	Fluazinam	Pyraclostrobin
*S. sclerotiorum* SC382	0.2017 ± 0.0069 c ^1^	0.0024 ± 0.0001 a	0.1317 ± 0.0069 c
*S. sclerotiorum* SM4	0.1057 ± 0.0063 a	0.0024 ± 0.0003 a	0.0908 ± 0.002 a
*S. nivalis* SM8	0.1908 ± 0.0015 b	0.0107 ± 0.0007 b	0.1175 ± 0.002 b

^1^ Different letters indicate a significant difference in values, according to Duncan’s test, at *p* = 0.05. All tests were carried out three times. The standard deviation (SD) is shown for each value.

## Data Availability

The original contributions presented in this study are included in the article/Supplementary Material. Further inquiries can be directed to the corresponding author.

## Supplementary Materials

The following supporting information can be downloaded at: https://www.mdpi.com/article/10.3390/plants14223487/s1, Figure S1: The colony diameter of *Sclerotinia sclerotiorum* (strains SC382 and SM4) and *S. nivalis* (strain SM8) at different boscalid concentrations after 3 days of cultivation. Table S1: List of genomic assemblies used for MLST phylogeny and ANI analysis. Table S2: Sample sizes (n), normality and homogeneity tests, exact *p*-values, and clarified the design of ANOVA. File S1: Draft genome assembly of *Sclerotinia nivalis* strain SM8.
