# Supplementary material for: First Detection of Sclerotinia nivalis on Carrot (Daucus carota subsp. sativus) in Russia and Comparative Pathogenicity of Sclerotinia Isolates on Carrot
Source: Plants (Basel). 2025 Nov 15;14(22):3487. doi: 10.3390/plants14223487 (PMC12655907; doi:10.3390/plants14223487)
Supplement: Supplementary file 1 [file plants-14-03487-s001.zip › Supplementary Figure S1.pdf]

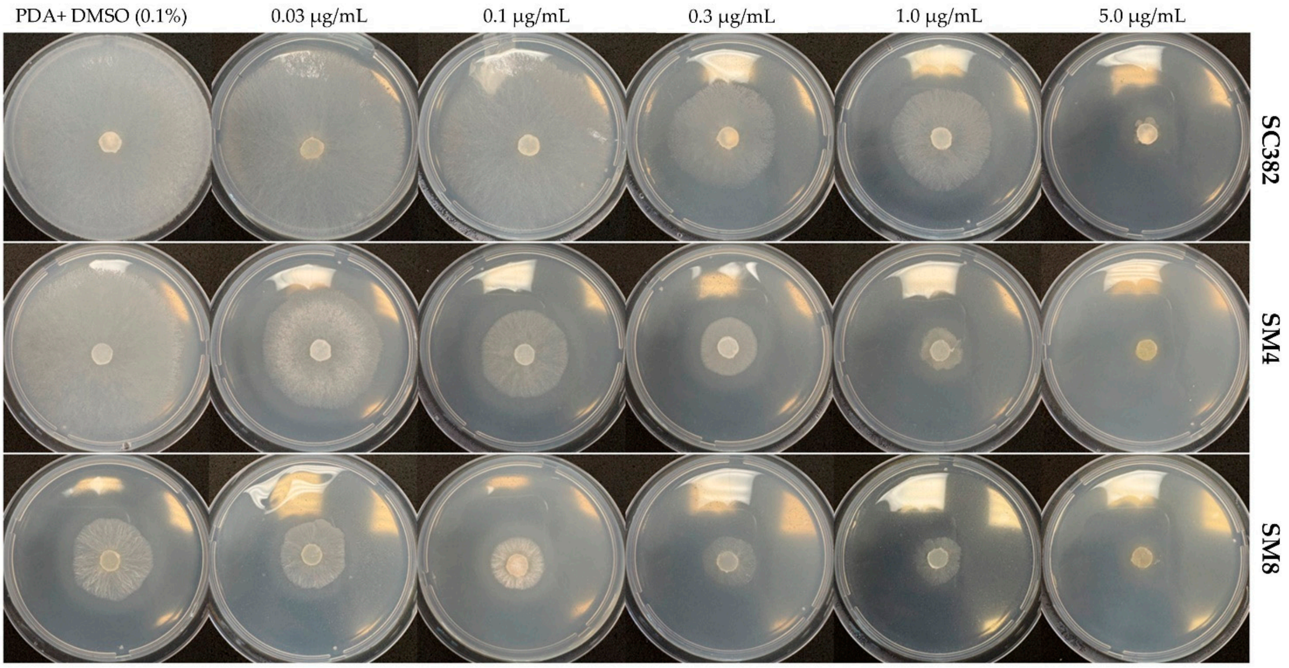

Supplementary Figure S1. The colony diameter of *Sclerotinia sclerotiorum* (strains SC382 and SM4) and *S. nivalis* (strain SM8) at different boscalid concentrations after 3 days of cultivation
