# Supplementary material for: First Detection of Sclerotinia nivalis on Carrot (Daucus carota subsp. sativus) in Russia and Comparative Pathogenicity of Sclerotinia Isolates on Carrot
Source: Plants (Basel). 2025 Nov 15;14(22):3487. doi: 10.3390/plants14223487 (PMC12655907; doi:10.3390/plants14223487)
Supplement: Supplementary file 1 [file plants-14-03487-s001.zip › Supplementary Table S2.pdf]

Supplementary Table S2. Sample sizes (n), normality and homogeneity tests, exact p-values, and clarified the design of ANOVA.

Parameter (Figure/Table number)	n	Shapiro–Wilk p	Levene p	ANOVA p	Duncan groups
Radial growth rate (Fig. 5C)	4	0.365 - 0.91	0.41	<0.0001	SC382 (a), SP1 (a), SM8 (b), SM4 (c)
Sclerotia number (Fig. 5D)	4	0.135 - 0.969	0.450	$3.0×10^{-7}$	SM8 (a), SP1 (b), SC382 (c), SM4 (d)
Total sclerotia mass (Fig. 5E)	4	0.257 - 0.777	0.250	$1.41×10^{-10}$	SC382 (a), SP1 (b), SM4 (b), SM8 (c)
Average sclerotium mass (Fig. 5F)	4	0.388 - 0.959	0.094	$2.0×10^{-17}$	SC382 (a), SM4 (b), SP1 (c), SM8 (d)
Lesion area on leaves, day 9 (Fig. 8A)	5	0.432 - 1.000	0.0686	$8.6×10^{-12}$	-
Lesion area on roots, day 9 (Fig. 8B)	5	0.213 - 1.000	0.0918	$2.18×10^{-6}$	-
EC50 boscalid (Table 2)	3	0.0168 - 0.713	0.249	$1.38×10^{-7}$	SM4 (a), SM8 (b), SC382 (c)
EC50 fluazinam (Table 2)	3	0.228 - 0.978	0.907	$1.21×10^{-9}$	SM4 (a), SC382 (a), SM8 (b)
EC50 pyraclostrobin (Table 2)	3	0.560 - 0.879	0.274	$3.11×10^{-7}$	SM4 (a), SC382 (c), SM8 (b)
